# Lockdown measures during the COVID-19 pandemic strongly impacted the circulation of respiratory pathogens in Southern China

**DOI:** 10.1038/s41598-022-21430-x

**Published:** 2022-10-08

**Authors:** Heping Wang, Yuejie Zheng, Marien I. de Jonge, Rongjun Wang, Lilly M. Verhagen, Yunsheng Chen, Li Li, Zhi Xu, Wenjian Wang

**Affiliations:** 1 Shenzhen Children’s Hospital, No. 7019 Yitian Road, Futian District, Shenzhen, 518038 Guangdong China; 2 Department of Laboratory Medicine, Laboratory of Medical Immunology, Radboud Center for Infectious Diseases, Radboud University Medical Center, Nijmegen, The Netherlands; 3 Department of Pediatric Infectious Diseases and Immunology, Amalia Children’s Hospital, Radboud University Medical Center, Nijmegen, The Netherlands; 4 Ningbo Health Gene Technologies Co., Ltd, Ningbo, Zhejiang China

**Keywords:** Microbiology, Pathogens

## Abstract

A range of public health measures have been implemented to suppress local transmission of coronavirus disease 2019 (COVID-19) in Shenzhen. We examined the effect of these measures on the prevalence of respiratory pathogens in children. Clinical and respiratory pathogen data were collected for routine care from hospitalized children with acute respiratory infections in Shenzhen Children’s Hospital from July 2018 to January 2022. Nasopharyngeal swabs were collected and respiratory pathogens were detected using standardized clinical diagnostics as part of routine care. Data were analyzed to describe the effects of COVID-19 prevention procedures on other common pathogens. A total of 56,325 children under 14 years of age were hospitalized with an acute respiratory infection during the study period, 33,909 were tested from July 2018 to January 2020 (pre-lockdown), 1168 from February 2020 to May 2020 (lockdown) and 21,248 from July 2020 to January 2022 (post-lockdown). We observed a 37.3% decline of routine care in respiratory infection associated hospital admission in the 19 months’ post-lockdown vs. the 19 months’ pre-lockdown. There were 99.4%, 16.0% and 1.26% reductions measured for *Mycoplasma pneumoniae*, influenza virus A and adenovirus, respectively. However, a 118.7% and 75.8% rise was found for respiratory syncytial virus (RSV) and human para-influenza virus (HPIV) during the 19 months’ post-lockdown in comparison to the pre-pandemic period. The detection of RSV especially increased in toddlers after the lockdown. Lockdown measures during the COVID-19 pandemic led to a significant reduction of *Mycoplasma pneumoniae,* influenza virus A and adenovirus infection. In contrast, RSV and HPIV infection increased.

## Introduction

Coronavirus disease 2019 (COVID-19), caused by severe acute respiratory syndrome coronavirus 2 (SARS-CoV-2), was first detected in December 2019 in Wuhan. The World Health Organization (WHO) declared the outbreak to be a pandemic on March 11, 2020. Subsequent implementation of non-pharmaceutical interventions (e.g. cessation of global travel, mask use, regular hand-sanitizing and hand-washing, physical distancing, avoid gatherings, and staying home) reduced transmission of SARS-CoV-2^1–6^. It is observed that SARS-CoV-2 measures may also be effective in reducing acute respiratory infectious (ARIs) diseases, such as seasonal influenza, outpatient pneumonia^7–10^.

Shenzhen is a large migratory city located in southern China, north of Hong Kong. Shenzhen, like most domestic cities in China, has implemented strict COVID-19 prevention and control measures from February 2022. For specific measures, including the wearing of masks, social distancing, and avoidance of gathering-related activities, refer to the paper published by our group^11^. Public health measures, such as wearing masks, washing hands, and disinfecting hands with alcohol are effective against various infectious diseases, including rotavirus, norovirus and respiratory infections^10,12–15^.

Influenza virus is one of the most concerned seasonal pathogens, a reduction in the number of people infected with the influenza virus in 2020 compared to the previous year was observed in studies from Japan, France, Singapore, Mexico, Hong Kong and Zhejiang province in China^16–21^. We have also previously reported a significant reduction in the incidence of respiratory pathogens post-lockdown, however this measures in a different period, only from September to December (2020) and not year round as described in this study^11^. Current infection control measures for COVID-19 impacted the respiratory infections focused on the effects of Center for Disease Control and Prevention (CDC) population-wide data^5,13,22^, and only a few studies have focused on children over long periods of time^9^. Here, we assessed the change of the seasonal influenza virus incidence and respiratory pathogen infections pre- and post-lockdown in hospitalized children for 42 months.

## Materials and methods

### Patients information

Patients with ARI between July 2018 and January 2022 admitted to the pediatric wards were enrolled in Shenzhen Children’s Hospital. The inclusion criteria were as follows: age below 14 years and one or more respiratory symptoms involving the respiratory tract (rhinorrhea, sore throat, cough, dyspnea/tachypnea), SARS-CoV-2 were not detected since January 2020. The exclusion criteria were as follows: repeated the detection within one week, the patient was over 14 years old, and the age or gender information was incomplete. Laboratory and demographic data of the patients enrolled in this study were retrieved from the Shenzhen Children’s Hospital electronic patient dossiers. The study protocol was approved and waived informed consent by the Ethical Committee of Shenzhen Children’s Hospital (201601304). The study was performed in accordance with relevant guidelines and regulations.

### Statistical analyses

We compared the overall number of hospitalized children before and after the lockdown measures, described by monthly distribution. Paired *t*-test was performed according to the monthly number of children to observe the differences of hospitalized children pre- and post-lockdown. At the same time, the age composition ratio of hospitalized children was observed in pre- and post-lockdown. We compared age-group hospitalization rates for respiratory pathogens for the 19 months’ post-lockdown measures at the end of January 2022 with rates from the pre-lockdown 19 months.

The overall detection rate and prevalence of common pathogens, including respiratory syncytial virus (RSV), adenovirus (AdV), influenza A/B (InfA/B) and parainfluenza virus (HPIV, types 1, 2 and 3) and *M. pneumoniae* (*Mp*), were compared pre-and post-lockdown periods. Differences in detection methods were also taken into account when comparing. At the same time, to observe the seasonal prevalence of the pathogens, including human metapneumovirus (HMPV), human rhinovirus (HRV), human bocavirus (HBoV), human coronavirus (HCOV, 229E, OC43, NL63 and HKU1), *Chlamydophila pneumoniae* (*Cp*), expanded by the GeXP-based multiplex reverse transcription polymerase chain reaction (PCR) assay started in September 2019. We compared PICU admission and respiratory pathogens detection rates between the pre-lockdown and post-lockdown period and whether it was consistent with the overall prevalence.

SPSS 21.0 was used to analyze difference in patient number and pathogen prevalence with 95% confidence intervals. Descriptive statistics and paired *t*-test were applied to describe patient characteristics and difference. Categorical variables were analyzed by Pearson's chi-squared test. *P* < 0.05 was considered statistically significant.

## Results

### Patient characteristics

**Figure 1:**
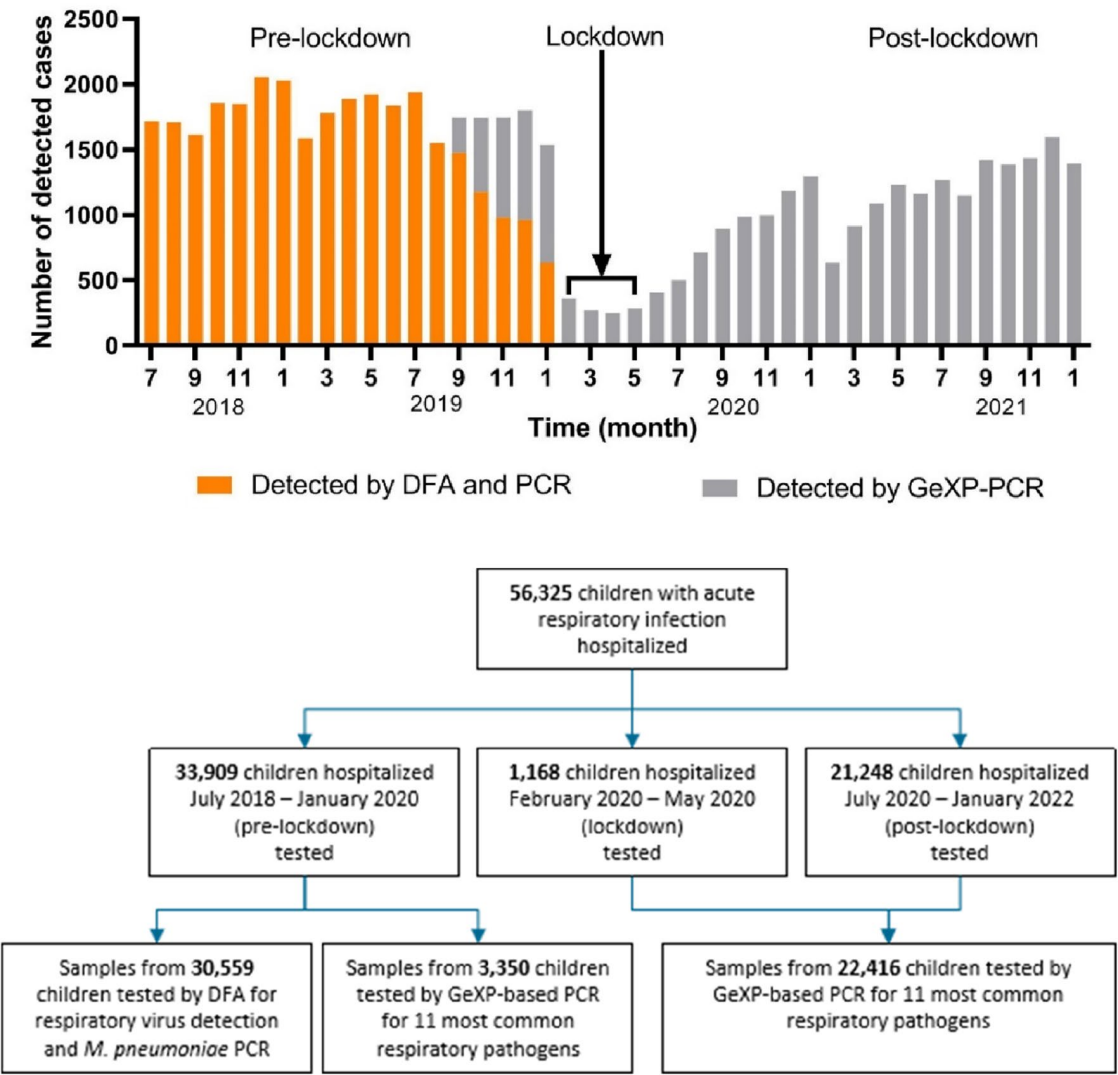
Number of detected cases by month (above) and the flowchart (below) of detection by DFA or GeXP-based PCR. Specimens were tested by DFA and *M. pneumoniae* PCR in pre-lockdown (orange bar), and tested by GeXP-based multiplex reverse transcription PCR assay (grey bar). DFA, Direct Immunofluorescence Assay; pre-lockdown, from July 2018 to January 2020, Lockdown, from February to May 2020, post-down, from June 2020 to January 2022.

**Figure 2:**
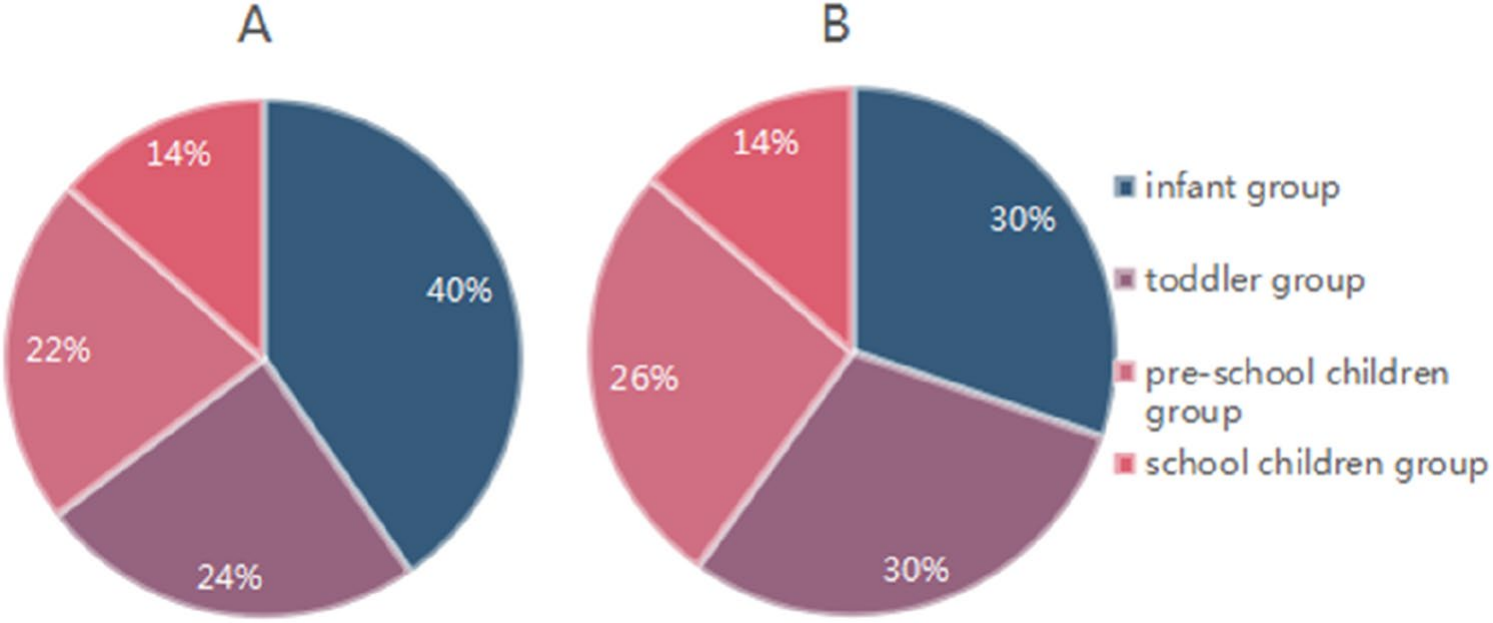
The proportion of each group in pre- and post-lockdown. Pre-lockdown (**A**), from July 2018 to January 2020, post-down (**B**), from July 2020 to January 2022.

A total of 56,325 children with ARIs were enrolled in this study, 33,909 were tested from July 2018 to January 2020 (pre-lockdown), 1168 from February to May 2020 (lockdown) and 21,248 from July 2020 to January 2022 (post-lockdown), indicating a 37.3% reduction in hospital admissions due to ARI comparing the post-lockdown versus the pre-lockdown period. The number of monthly hospitalized children for ARIs did not return to the pre-lockdown number in the 20 months following the lockdown (Fig. 1). The median number of hospitalizations per month of children were 1784 before and reduced to 1,118 after the lockdown. There was a significant difference in the number of patients monthly between the two groups in pre- and post-lockdown (*t* = 9.859, *P* < 0.001). To determine age specific differences in viral infections before and after the lockdown period, we divided the patients according to age into four groups, as follows (1) infants (newborn-1 year old), (2) toddlers (1–3 years old), (3) pre-school children (3–6 years old), (4) school children (6–14 years old); the proportion of hospitalized children in each group before and after the lockdown is shown in Fig. 2A,B. There was a significant difference in age groups between pre- and post-lockdown (*p* < 0.001). There was no significant difference in sex ratio (*p* = 0.8041).

### The prevalence of respiratory pathogens in the pre- and post-lockdown period

In total 30,559 specimens collected were tested by DFA and *Mp* PCR from July 2019 to January 2020 and 3350 specimens were tested by GeXP-based multiplex reverse transcription PCR assay from September 2019 to January 2020. All specimens collected during and after the lockdown (22,416) were tested by GeXP-based multiplex reverse transcription PCR assay as depicted in Fig. 1. The detection rates of *Mp,* RSV, AdV, HPIV, InfA and InfB before the lockdown were 16.9%, 6.3%, 2.7%, 1.8%, 1.3% and 0.2%, respectively. The most frequently detected pathogens after the lockdown were HRV (26.7%), RSV (18.2%), HPIV (8.6%), HMPV (5.1%), AdV (2.6%), *Mp* (1.4%), followed by HBoV (2.4%), HCOV (1.9%), InfB (1.5%), InfA (0.03%) and *Cp* (0.6%).

**Figure 3:**
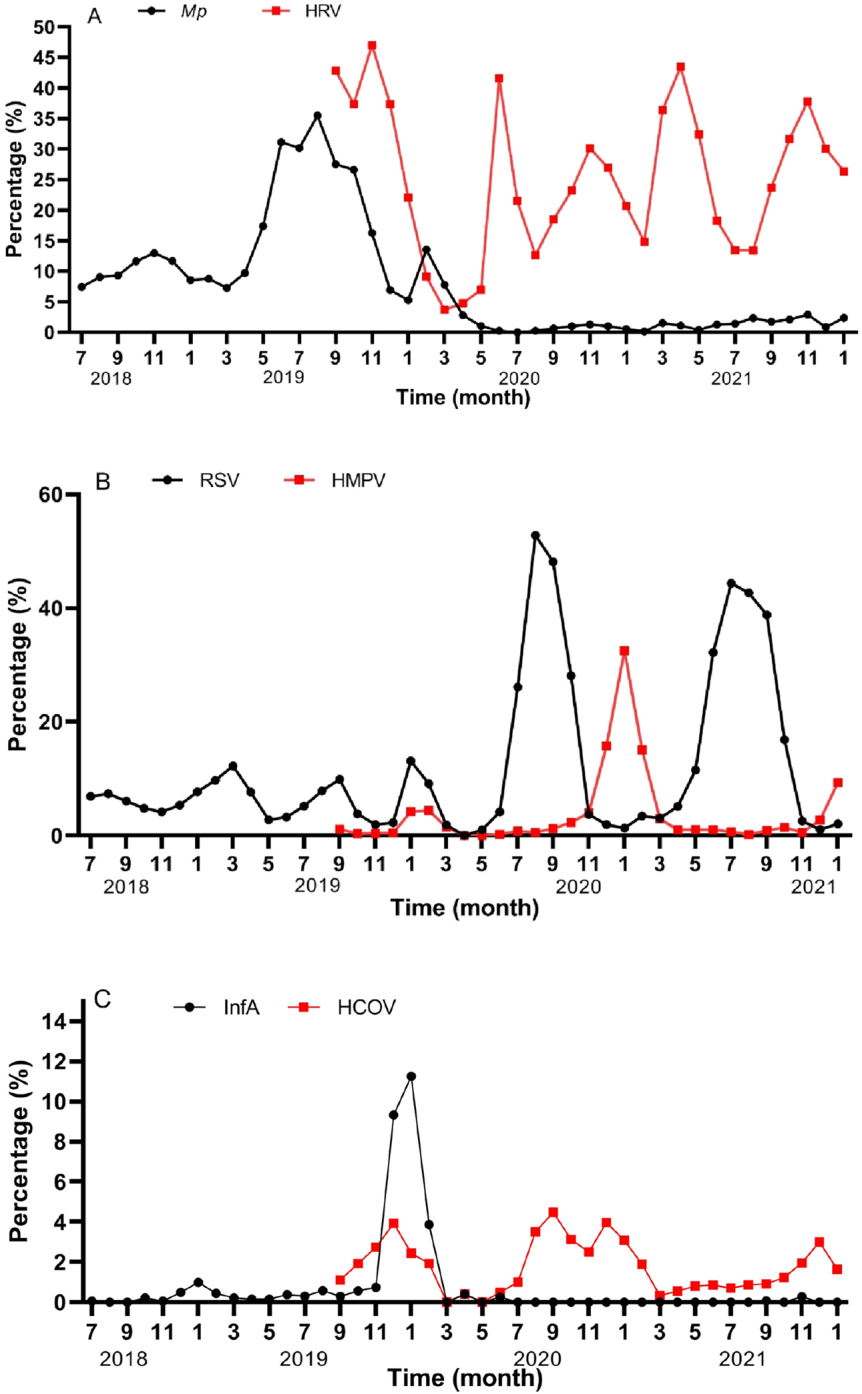

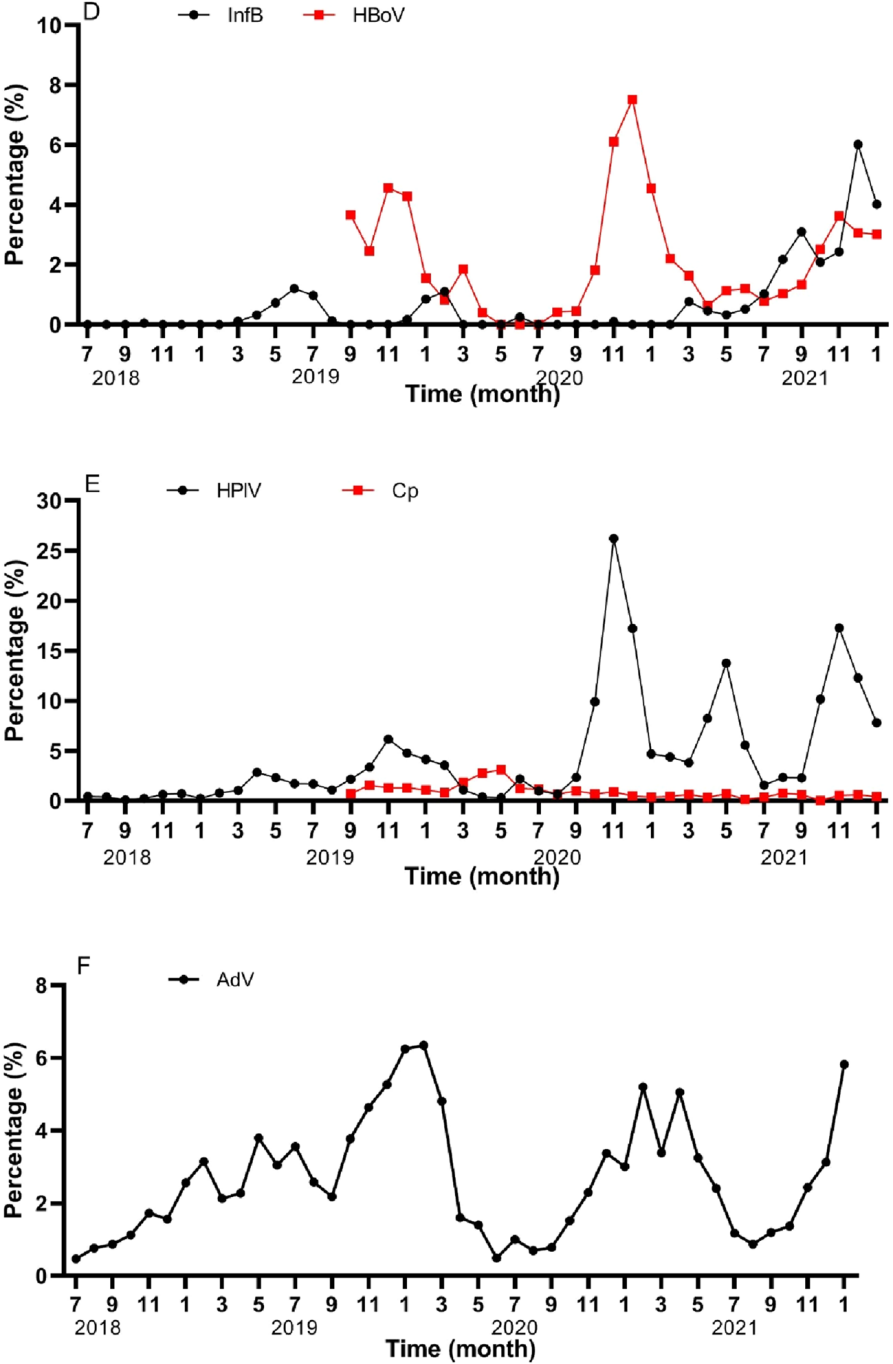
The seasonal prevalence of 11 respiratory pathogens (**A**–**F**). *Mycoplasma pneumoniae* (*Mp*), HRV, RSV, HMPV, influenza A, HCOV, influenza B, HBoV, HPIV and AdV the percentage detected was plotted as a function of time (indicated in months) before, during or after lockdown. Black line represented *Mp*, RSV, InfA, InfB, HPIV and AdV in (**A**–**F**) from July 2018 to January 2022, red line was HRV, HMPV, HCOV, HBoV, *Cp* in A-E from September 2019 to January 2022. AdV, adenovirus; HRV, human rhinovirus; HPIV, human parainfluenza viruses 1–4; RSV, respiratory syncytial virus; HMPV, human metapneumovirus; HCOV, human coronavirus; InfA/B, influenza A/influenza B; HBoV, human bocavirus; *Cp, Chlamydophila pneumoniae*.

We determined the prevalence of the six major pathogens including *Mp*, InfA, InfB, AdV, RSV, and HPIV before and after the lockdown period. It was found that the detection rate of InfA and *Mp* decreased significantly during and after the lockdown, the detection rate of *Mp* decreased from 15 to 40% to about 1%, while infection rates with RSV and HPIV were consistent in the pre- and post-lockdown period (Fig. 3A–F). AdV showed a decrease during the lockdown, however the number of infections quickly increased after the lockdown.

The lockdown measures clearly distorted the common seasonal dynamics of respiratory infections. In Southern China, InfA peaks every year in January and normally there is also a small peak in August^21^, these seasonal patterns completely disappeared. *Mp* has normally a high detection rate throughout the year, as was seen especially in 2019. RSV peaked in both summer and autumn in the pre-lockdown and post-lockdown period, however the peak in March during the post-lockdown period did not appear. HPIV prevalence showed the same trend pre- and post-and peaked in May and November. Also AdV in pre-lockdown and post-lockdown have the same trend, both are common in the winter and spring season. The transition between summer and autumn is the time with the lowest detection rates for this pathogen. InfB is normally sporadically detected, however the detection rate has increased from 0.5% in June 2021 to 4.0% in January 2022.

From September 2019, we have added HRV, HMPV, HBoV, HCOV and *Cp* to our PCR-based respiratory pathogen detection panel. This was used to determine the detection rate between September 2019 and January 2022. We found that the detection rate of HRV was above 10% throughout the year, with the highest detection rate in April and November each year. The detection rate of HMPV was lower throughout the year, but the highest detection rate was in January 2020 to 2022. HCOV has the highest detection rate in December 2020 to 2021, and HBoV is the highest in November and December; *Cp* is at a low level throughout the year, mainly detected in neonates and patients within 6 months, and in lockdown, *Cp* is the only pathogen with an increased detection rate (Fig. 3).

### Comparison of PICU admission and respiratory pathogens detection rates between the pre- and post-lockdown period

A total of 1366 (4.03%) and 817 (3.85%) children were admitted to the PICU, before and after the lockdown, respectively. The rate of admission to the PICU decreased after the lockdown, however there was no significant difference found compared to the pre-lockdown period (*p* > 0.05). The detection rates of AdV, *Mp* and InfA in children admitted to the PICU were reduced from 4.4%, 3.7% and 2.5% pre-lockdown to 0.7%, 0.2% and 0.5% post-lockdown. In contrast, HPIV and RSV were increased from 0.7% and 8.9% to 3.7% and 11.6%. We found the detection rate of HBoV reached 4.2% in post-lockdown, which was an important virus detected in PICU patients.

### Comparison of detection rates of respiratory pathogens by different ages and gender before and after the lockdown

**Figure 4:**
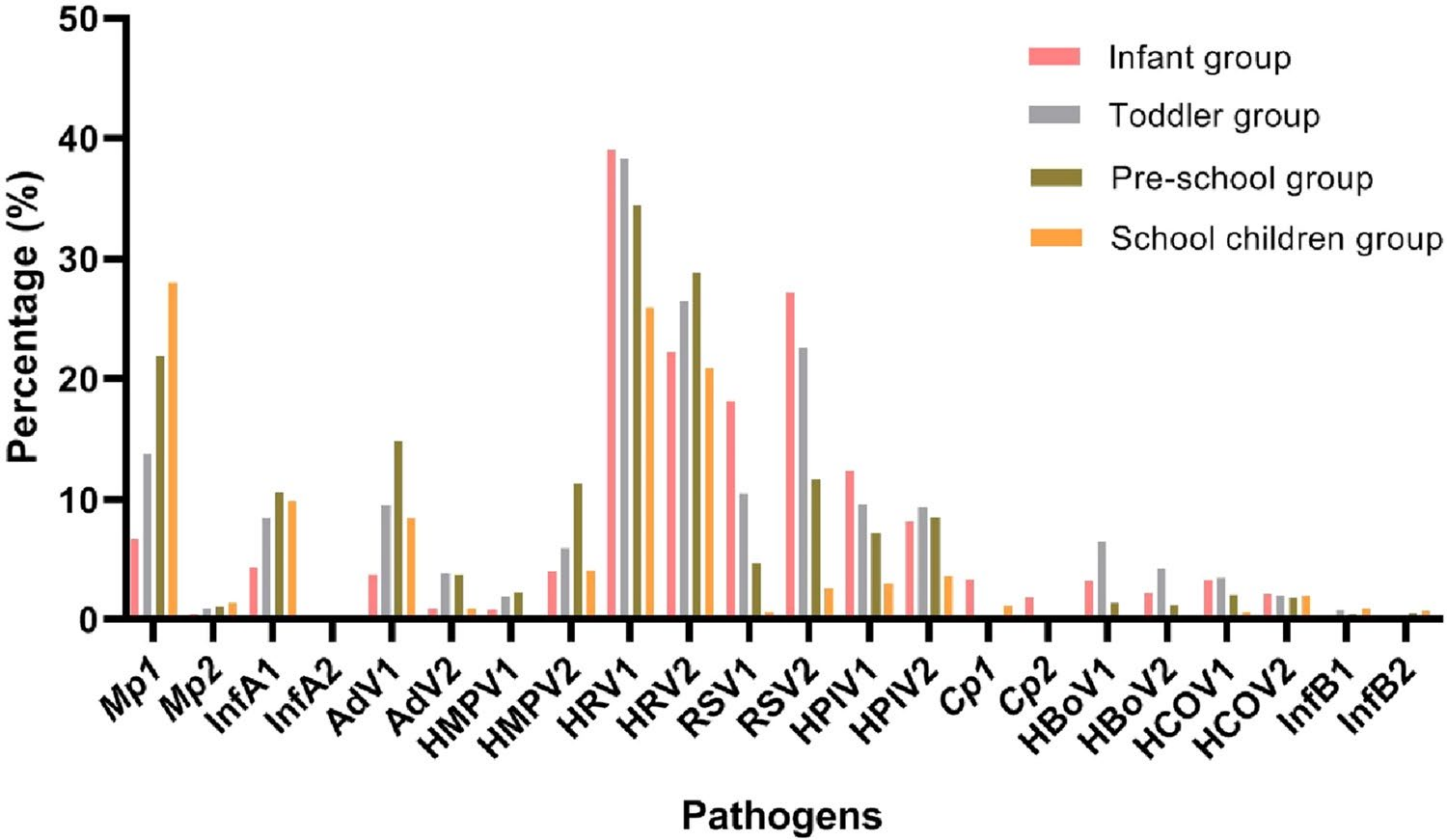
The distribution of respiratory pathogens at different age groups, and the differences pre-lockdown (1) and post-lockdown (2) were compared. AdV, adenovirus; HRV, human rhinovirus; HPIV, human parainfluenza viruses 1–4; RSV, respiratory syncytial virus; HMPV, human metapneumovirus; HCOV, human coronavirus; InfA/B, influenza A/influenza B; HBoV, human bocavirus; *Cp, Chlamydophila pneumoniae*.

Comparison of pre-lockdown and post-lockdown, the changes of detection rates of respiratory pathogens in each age group were relatively consistent, and only the detection rate of HRV in the infant group decreased significantly in post-lockdown. The detection rate of *Mp* increased with age. InfA, adenovirus and HMPV had the highest detection rate in the pre-school group and the lowest in the infant group. Rhinovirus, RSV, HPIV and *Cp* had the highest detection rate in the infant group. HBoV and HCoV had the highest detection rates in the toddler group (Fig. 4).

The detection rate of *Mp* in female patients in the pre-lockdown period was significantly higher than that of male patients (*p* < 0.001), but there was no difference post-lockdown (*p* = 0.6748), due to the significant decrease in the detection rate. In contrast, RSV was significantly more frequently detected in males than in females in post-lockdown (*p* = 0.0012), while no difference was observed before the lockdown (*p* = 0.0575).

## Discussion

Shenzhen is located in southern China, after the first case of COVID-19 imported from Hubei province on January 19, 2020, the strictest control measures (lockdown) were implemented from February to May 2020. Under the prevention and control of the public health measures the number of hospitalized children with ARIs has dropped significantly^20^. Only 16.3% of the total hospitalized patients in February to May 2019 were children with acute respiratory infections. This is likely due to the strict prevention and control measures, the medical treatment of children with respiratory diseases in local social health centers to prevent travelling with diseased children, and the decline in the number of people returning to Shenzhen from outside the province.

We had chosen for the time span covering the two influenza seasons before the lockdown. The detection rate of InfA decreased significantly post-lockdown, only very few positive samples were detected in the post-lockdown period until January 2022, which is consistent with other studies^8,16–18,20–23^. Also other pathogens, such as *Mp* and AdV, which are predominant in older children, were also significantly reduced. Interestingly, it was found that the prevalence of RSV and HPIV increased after the lockdown. It may be due to a phenomenon called viral interference where one virus blocks the growth of another virus^11,24^. Although this is a limitation of the study, caused by the fact that inclusion was based on routine diagnostics and thus dependent on the implemented diagnostic methods, the increase is found for multiple but not all viruses.

Public health measures implemented during the COVID-19 pandemic strongly impacted the incidence of common respiratory pathogens^1,3,16,25,26^. InfA almost disappeared. This may be related to the fact that people wear masks and maintain social distance. It might also be related to the fact that Shenzhen has carried out influenza target group vaccination before December 2020 in order to prevent the double epidemic of influenza and SARS-CoV-2. The detection rate of *Mp* decreased from 10 to 40% to about 1%, which may be related to the annual change of its prevalence as a consequence of the public health measures. RSV did not appear after the peak detection rate in March, and the peak in summer and autumn in the first year after the pandemic of COVID-19 was delayed by about a month compared to the second year. However, it may also be related to the subtype of the epidemic strain, as well as to the climatic conditions in different years.

Severe respiratory tract infection is an important cause of death in children, and it is of great significance to explore respiratory pathogens in children admitted to the PICU. In this study, under the general public health measures in the post-lockdown period, the proportion of children admitted to the PICU was the same as before the lockdown. However, we also found that the detection rate of respiratory pathogens in the PICU was consistent with the overall epidemiological detection rate in the study period. It should be pointed out that in post-lockdown, we used PCR detection to increase the detection of various viruses, and found that the detection rate of HBoV in children admitted to PICU reached 4.2%, which is a very important pathogen. However, more research is needed to understand the role of HBoV in children with severe respiratory infections.

## Data Availability

The key information and data generated and/or analyzed during this study were included in this article.

## Abbreviations

InfA: Infuenza A
InfB: Infuenza B
HPIV: Human parainfuenza virus
RSV: Respiratory syncytial virus
AdV: Adenoviruses
HMPV: Human metapneumovirus
HRV: Human rhinovirus
HBoV: Human bocavirus
HCoV: Human coronavirus
Ch: Chlamydia
MP: Mycoplasma pneumoniae
PCR: Polymerase chain reaction
